# Association between free fatty acids and adverse outcomes in patients with and without diabetes undergoing percutaneous coronary intervention

**DOI:** 10.1515/jtim-2026-0016

**Published:** 2026-02-13

**Authors:** Qinxue Li, Guyu Zeng, Deshan Yuan, Tianyu Li, Peizhi Wang, Ce Zhang, Sida Jia, Pei Zhu, Ying Song, Xiaofang Tang, Ping Liu, Yuejin Yang, Runlin Gao, Jingjing Xu, Xueyan Zhao, Jinqing Yuan

**Affiliations:** Department of Cardiology, Xuanwu Hospital, Capital Medical University, National Clinical Research Centre for Geriatric Diseases, Beijing, China; Department of Cardiology, National Clinical Research Center for Cardiovascular Diseases, State Key Laboratory of Cardiovascular Disease, Fuwai Hospital, National Center for Cardiovascular Diseases, Chinese Academy of Medical Sciences and Peking Union Medical College, Beijing, China

**Keywords:** free fatty acids, coronary artery disease, percutaneous coronary intervention, diabetes mellitus, major adverse cardiovascular and cerebrovascular events

## Abstract

**Background and Objectives:**

This study aimed to explore the correlation between free fatty acid (FFA) levels and adverse outcomes in patients undergoing percutaneous coronary intervention (PCI) with or without diabetes mellitus.

**Methods:**

In total, 10,230 patients treated with PCI were included in this study and divided into three equal groups according to FFA levels (FFA-L, FFA-M, and FFA-H groups). Subsequently, the patients were further stratified based on their diabetes status. A 5-year follow-up was conducted, with the primary endpoint defined as major adverse cardiovascular and cerebrovascular events (MACCE).

**Results:**

During follow-up, 2108 (20.6%) patients experienced MACCE. In patients without diabetes, no significant difference was observed in the risk of MACCE among the different FFA groups. However, in patients with diabetes, the risk of MACCE was significantly higher in the FFA-L and FFA-H groups than in the FFA-M group [adjusted hazard ratio (HR), 1.238, 95% confidence interval (CI), 1.054–1.454, *P* = 0.009; adjusted HR: 1.220, 95% CI, 1.054–1.412, *P* = 0.008; respectively]. The restricted cubic spline curves showed a nonlinear U-shaped relationship between the FFA levels and the risk of MACCE in patients with diabetes, with the lowest risk observed at an FFA level of 372 μmol/L. The results of the subgroup analysis stratified by different clinical presentations and BMI were similar to those of the primary findings.

**Conclusions:**

In patients with diabetes undergoing PCI, both elevated and decreased FFA levels were significantly associated with an increased risk of MACCE. Monitoring FFA levels is essential to help identify those at high risk.

## Introduction

Free fatty acids (FFAs), also known as non-esterified fatty acids, are released into the circulation from the adipose tissue through triglyceride (TG) lipolysis.^[1]^ FFAs are sensitive indicators of changes in lipid metabolism. In healthy individuals, FFAs provide approximately 70% of the energy required for myocardial metabolism.^[2]^ Elevated FFA levels can induce endothelial dysfunction and increase inflammatory responses, thereby contributing to the development of atherosclerosis.^[3,4]^ High FFA levels can promote arrhythmias and are closely associated with sudden cardiac death.^[5,6]^ Elevated baseline FFA levels are reportedly associated with an increased risk of death and adverse cardiovascular events in patients with coronary artery disease (CAD).^[7, 8, 9]^

A close relationship exists between FFA levels and diabetes mellitus (DM).^[10]^ High FFA levels are strongly associated with insulin resistance and impaired pancreatic β-cell function.^[11,12]^ Individuals with higher plasma FFA levels are at greater risk of developing diabetes.^[13,14]^ Reducing FFA levels can reportedly improve insulin sensitivity and decrease oxidative stress in patients with diabetes.^[15,16]^ However, limited and inconsistent research exists on the relationship between FFA levels and adverse outcomes in patients with CAD with different diabetes statuses. Jin *et al*. demonstrated that in patients with stable CAD and diabetes, the increased FFA levels was associated with poor prognosis.^[17]^ However, the research conducted by Pan *et al*. revealed a U-shaped relationship between FFA levels and adverse outcomes in patients with CAD and diabetes.^[18]^ These findings limit the ability to predict the risk of cardiovascular events based on FFA levels, particularly in patients with both diabetes and CAD. Therefore, our study aimed to investigate the correlation between FFA levels and adverse outcomes in patients undergoing percutaneous coronary intervention (PCI) with different diabetes statuses, utilizing a larger sample size and a longer follow-up period.

## Methods

### Research design

**Figure 1:**
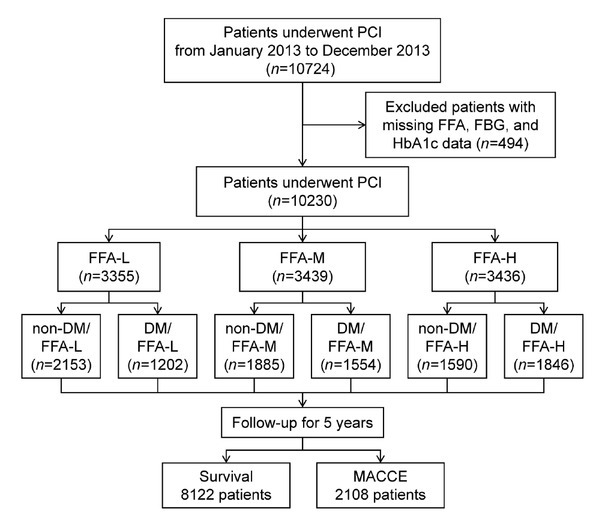
Flowchart of the study. PCI: percutaneous coronary intervention; FFA: free fatty acid; FBG: fasting blood glucose; HbA1c: glycated hemoglobin; DM: diabetes mellitus; MACCE: major adverse cardiovascular and cerebrovascular events.

From January to December 2013, 10,724 patients with CAD undergoing PCI were consecutively enrolled at Fuwai Hospital, Chinese Academy of Medical Sciences. Inclusion criteria were a diagnosis of CAD and undergoing PCI during hospitalization, with consent for follow-up. After excluding patients with missing data on FFA (*n* = 300), glycated hemoglobin (HbA1c) (*n* = 192), and fasting blood glucose (FBG) (*n* = 2), the study ultimately included 10,230 CAD patients treated with PCI (Figure 1). Fasting blood samples were collected within 24 h of admission, and FFA levels were measured using an automatic biochemical analyzer (Hitachi 7150, Tokyo, Japan). Patients were divided into three equal groups based on FFA levels: FFA-L (FFA < 320 μmol/L) (*n* = 3355), FFA-M (320 μmol/L ≤ FFA < 480 μmol/L) (*n* = 3439), and FFA-H (FFA ≥ 480 μmol/L) (*n* = 3436). Further, patients were divided into six groups based on FFA levels and diabetes status: non-DM/FFA-L (*n* = 2153), non-DM/FFA-M (*n* = 1885), non-DM/FFA-H (*n* = 1590), DM/FFA-L (*n* = 1202), DM/FFA-M (*n* = 1554), DM/FFA-H (*n* = 1846). General clinical data, laboratory test results, and coronary interventional treatment information were collected from the medical and interventional records of the patients. This study complied with the Declaration of Helsinki and the ethical standards of Fuwai Hospital and was approved by the Fuwai Hospital Review Committee. Because this study is a retrospective study, informed consent was waived.

### Procedure and medications

PCI strategy and stent type were determined by the interventional cardiologist based on the condition of the patient. Patients undergoing elective PCI, unless on long-term P2Y12 inhibitor therapy, received oral aspirin (300 mg) and clopidogrel (loading dose, 300 mg) or ticagrelor (loading dose, 180 mg) at least 24 h before the procedure. Patients with acute coronary syndrome (ACS) scheduled for PCI received the same doses of aspirin and ticagrelor or clopidogrel (loading dose, 300 or 600 mg) as soon as possible. During the procedure, all patients were administered heparin (100 U/kg), with glycoprotein IIb/IIIa inhibitors at the discretion of the interventional cardiologist. After the procedure, the patients were prescribed aspirin 100 mg daily and either clopidogrel 75 mg daily or ticagrelor 90 mg twice daily for at least 12 months.

### Follow-up and endpoint events

Follow-up data were obtained at five time points (1 month, 6 months, 1 year, 2 years, and 5 years post-discharge) through outpatient visits, telephone interviews, and correspondence. The primary endpoint was a composite endpoint defined as major adverse cardiac and cerebrovascular events (MACCE), including all-cause death, myocardial infarction (MI), unplanned revascularization, and ischemic stroke.^[19]^ The secondary endpoints were the individual components of MACCE. All the events were carefully reviewed and validated by a group of independent clinicians.

### Definitions

Diabetes was defined as FBG ≥ 7.0 mmol/L, HbA1c level ≥ 6.5%, 2-hour plasma glucose ≥ 11.1 mmol/L in an oral glucose tolerance test, current use of antidiabetic medication, or a previous diagnosis of diabetes. Left main coronary artery disease was defined as ≥ 50% stenosis of the left main coronary artery confirmed by coronary angiography. Triple-vessel disease (TVD) was defined as ≥ 50% stenosis in all three major coronary arteries (left anterior descending, circumflex, and right coronary artery). Successful revascularization was defined as residual stenosis < 30% and TIMI (thrombolysis in myocardial infarction) flow grade 3 at the end of the PCI procedure, based on visual estimation from angiography.

### Statistical analysis

Continuous variables were presented as mean ± standard deviation, and intergroup comparisons were made using ANOVA or Kruskal-Wallis *H* tests. Categorical variables were expressed as frequencies (percentages) and compared using chi-square or Fisher’s exact tests. Kaplan-Meier survival curves were constructed, and log-rank tests were used to compare the incidence of endpoint events between different groups. Univariable and multivariable Cox regression models were used to analyze hazard ratios (HR) and 95% confidence intervals (CI) for risk factors associated with endpoint events. The confounding factors adjusted in the multivariable Cox regression model included clinically relevant variables or those that showed statistical significance in the univariate Cox regression analysis (Supplementary Table S1). These factors included gender, age, previous MI, previous PCI, previous coronary artery bypass grafting (CABG), hypertension, hyperlipidemia, cerebrovascular disease (CVD), chronic obstructive pulmonary disease (COPD), creatinine, blood urea nitrogen (BUN), albumin, high-sensitivity C-reactive protein (hsCRP), left ventricular ejection fraction (LVEF), synergy between PCI with taxus and cardiac surgery (SYNTAX) score, TVD, successful PCI, and calcium channel blocker (CCB) at discharge. In subgroup analyses restricted to diabetic patients, additional diabetes-related covariates (duration, therapy) were further included. Restricted cubic spline analysis was used to visualize the relationship between continuous FFA levels and the risk of outcome events. Given that FFA levels are influenced by stress conditions and obesity, subgroup analyses were further conducted based on different clinical presentations and body mass index (BMI) levels. Finally, to evaluate the potential impact of missing data and bolster the robustness of our conclusions, we performed a sensitivity analysis using median imputation for missing FFA, HbA1c, and FBG values. Statistical analyses were performed using IBM SPSS 26.0 and R software (version 4.2.2), with a two-tailed *P*-value < 0.05 considered statistically significant.

## Results

### High FFA linked to adverse clinical and angiographic profiles

The baseline characteristics of all patients categorized according to FFA levels are shown in Table 1. Patients in the FFA-H group were older, had a higher proportion of females, and higher BMI and incidence of ACS. They also had a higher prevalence of hypertension, diabetes, and family history of CAD, but a lower incidence of previous MI and current smoking rates. Patients in the FFA-H group had higher levels of low-density lipoprotein cholesterol (LDL-C), high-density lipoprotein cholesterol (HDL-C), TG, FBG, HbA1c, albumin and hsCRP, but lower BUN levels. The proportion of patients with LVEF < 50% was also higher in this group. Coronary angiography results showed a higher proportion of TVD and higher SYNTAX scores in the FFA-H group. Regarding discharge medications, the FFA-H group had a higher rate of calcium channel blocker use. The baseline characteristics of the patients without diabetes (Supplementary Table S2) and those with diabetes (Supplementary Table S3) were comparable to those of the total population.

**Table 1:** Baseline characteristics of different FFA groups

Variables	FFA-L (*n* = 3355)	FFA-M (*n* = 3439)	FFA-H (*n* = 3436)	*P* value
Demographic characteristics				
Age (years)	58.24 ± 10.07	57.85 ± 10.30	59.02 ± 10.45	< 0.001^*^
Male	2695 (80.3)	2720 (79.1)	2479 (72.1)	< 0.001^*^
BMI (kg/m^2^)	25.30 ± 3.00	26.19 ± 3.13	26.31 ± 3.33	< 0.001^*^
Clinical presentation				
SAP	1374 (41.0)	1412 (41.1)	1296 (37.7)	0.006^*^
ACS	1981 (59.0)	2027 (58.9)	2140 (62.3)	
Coexisting conditions				
Previous MI	673 (20.1)	687 (20.0)	603 (17.5)	0.011^*^
Previous PCI	779 (23.2)	868 (25.2)	865 (25.2)	0.090
Previous CABG	125 (3.7)	140 (4.1)	148 (4.3)	0.473
Hypertension	1971 (58.7)	2262 (65.8)	2369 (68.9)	< 0.001^*^
Hyperlipidaemia	2199 (65.5)	2359 (68.6)	2316 (67.4)	0.026^*^
Family history of CAD	777 (23.2)	841 (24.5)	914 (26.6)	0.004^*^
CVD	384 (11.4)	364 (10.6)	345 (10.0)	0.168
PAD	95 (2.8)	80 (2.3)	96 (2.8)	0.349
COPD	80 (2.4)	75 (2.2)	80 (2.3)	0.845
DM	1202 (35.8)	1554 (45.2)	1846 (53.7)	< 0.001^*^
Current smoker	2024 (60.3)	2032 (59.1)	1795 (52.2)	< 0.001^*^
Laboratory measurements				
FFA (μmol/L)	222.87 ± 64.98	391.33 ± 45.83	670.60 ± 232.61	< 0.001^*^
HDL-C (mmol/L)	1.02 ± 0.26	1.00 ± 0.27	1.08 ± 0.30	< 0.001^*^
LDL-C (mmol/L)	2.41 ± 0.86	2.48 ± 0.90	2.63 ± 0.96	< 0.001^*^
TG (mmol/L)	1.59 ± 0.83	1.82 ± 1.01	1.93 ± 1.30	< 0.001^*^
FBG (mmol/L)	5.74 ± 1.74	6.07 ± 1.93	6.71 ± 2.39	< 0.001^*^
HbA1c	6.43 ± 1.10	6.63 ± 1.23	6.81 ± 1.35	< 0.001^*^
Creatine (μmol/L)	75.38 ± 14.97	75.35 ± 15.39	75.77 ± 16.96	0.476
BUN (mmol/L)	5.93 ± 1.67	5.84 ± 1.59	5.74 ± 1.72	<0.001^*^
Albumin (g/L)	41.76 ± 3.78	42.86 ± 3.96	44.05 ± 4.04	< 0.001^*^
hsCRP (mg/L)	2.81 ± 3.50	3.16 ± 3.73	3.68 ± 4.11	< 0.001^*^
Cardiac function				
LVEF < 50%	231 (6.9)	257 (7.5)	300 (8.7)	0.014^*^
Angiographic and procedural details				
Left main involved	225 (6.7)	199 (5.8)	230 (6.7)	0.204
TVD	1340 (39.9)	1439 (41.8)	1530 (44.5)	0.001^*^
SYNTAX score	11.56 ± 8.03	11.44 ± 7.98	12.07 ± 8.25	0.003^*^
Successful PCI	3220 (96.0)	3308 (96.2)	3283 (95.5)	0.391
Number of stents	1.78 ± 1.09	1.84 ± 1.13	1.79 ± 1.10	0.112
Medicine at discharge				
Aspirin	3317 (98.9)	3397 (98.8)	3389 (98.6)	0.676
Clopidogrel	3300 (98.4)	3385 (98.4)	3394 (98.8)	0.309
CCB	1560 (46.5)	1633 (47.5)	1792 (52.2)	< 0.001^*^
**β**-blocker	3000 (89.4)	3103 (90.2)	3127 (91.0)	0.088
Statin	3218 (95.9)	3315 (96.4)	3285 (95.6)	0.246
Nitrate	3287 (98.0)	3370 (98.0)	3356 (97.7)	0.585

Data are presented as *n* (%) or mean ± SD. *P < 0.05. FFA: free fatty acid; BMI: body mass index; SAP: stable angina pectoris; ACS: acute coronary syndrome; MI: myocardial infarction; PCI: percutaneous coronary intervention; CABG: coronary artery bypass grafting; CAD: coronary artery disease; CVD: cerebrovascular disease; PAD: peripheral vascular disease; COPD: chronic obstructive pulmonary disease; DM: diabetes mellitus; HDL-C: high-density lipoprotein cholesterol; LDL-C: low-density lipoprotein cholesterol; TG: triglyceride; FBG: fasting blood glucose; HbA1c: glycated hemoglobin; BUN, blood urea nitrogen; hsCRP: high-sensitivity C-reactive protein; LVEF: left ventricular ejection fraction; TVD: triple-vessel disease; SYNTAX: synergy between PCI with taxus and cardiac surgery; CCB: calcium channel blocker.

### FFA and diabetes status predict MACCE

**Figure 2:**
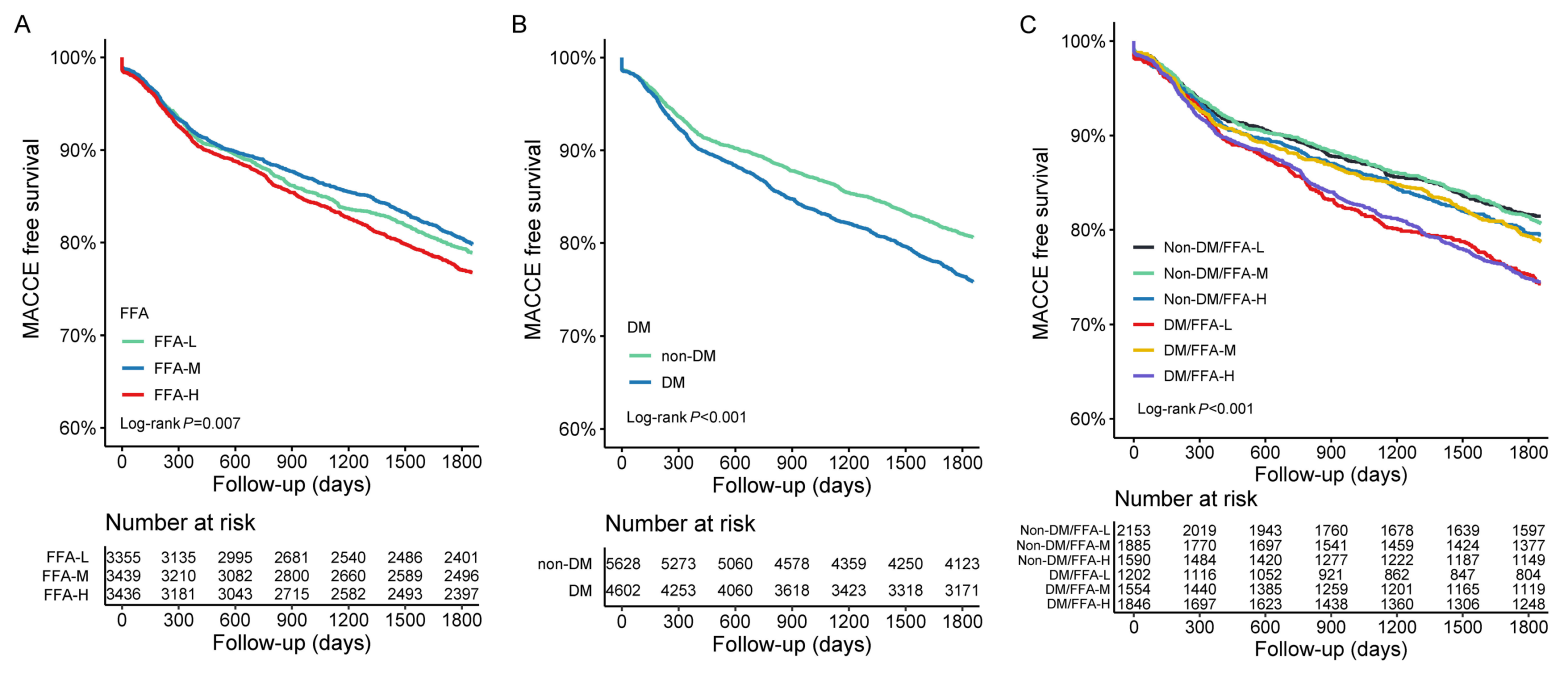
Kaplan-Meier analysis for MACCE according to FFA groups (A), diabetes status (B) and combined groups stratified by FFA and diabetes status (C). FFA: free fatty acid; DM: diabetes mellitus; MACCE: major adverse cardiovascular and cerebrovascular events.

During the 5-year follow-up period (interquartile range, 3.7-5.1 years), 2,108 (20.6%) MACCE were recorded. The FFA-M group exhibited the lowest incidence of MACCE (Table 2 and Figure 2A). The FFA-H group had a significantly higher risk of MACCE than the FFA-M group (adjusted HR, 1.131, 95% CI, 1.017–1.257; *P* = 0.023) (Table 2). Patients with diabetes had a significantly higher risk of MACCE than those without diabetes (adjusted HR, 1.144, 95% CI, 1.047–1.250; *P* = 0.003) (Table 2, Figure 2B).

**Table 2:** Univariate and multivariate Cox model analysis for MACCE for all participants

		Univariate analysis			Multivariate analysis		
Variables	Event/total (%)	Crude HR	95%CI	*P* value	Adjusted HR	95%CI	*P* value
FFA							
FFA-L	679/3355 (20.2)	1.058	0.950–1.177	0.305	1.085	0.973–1.209	0.141
FFA-M	663/3439 (19.3)	Ref	-	-	Ref	-	-
FFA-H	766/3436 (22.3)	1.177	1.061–1.306	0.002^*^	1.131	1.017–1.257	0.023^*^
Diabetes status							
Non-DM	1045/5628 (18.6)	Ref	-	-	Ref	-	-
DM	1063/4602 (23.1)	1.275	1.170-1.388	<0.001^*^	1.144	1.047-1.250	0.003^*^

* *P* < 0.05. The multivariate Cox model was adjusted for gender, age, previous myocardial infarction, previous percutaneous coronary intervention, previous coronary artery bypass grafting, hypertension, hyperlipidemia, cerebrovascular disease, chronic obstructive pulmonary disease, creatinine, blood urea nitrogen, albumin, high-sensitivity C-reactive protein, left ventricular ejection fraction, SYNTAX score, triple-vessel disease, successful previous percutaneous coronary intervention, and calcium channel blocker at discharge. FFA: free fatty acid; DM: diabetes mellitus; MACCE: major adverse cardiac and cerebrovascular events; HR: hazard ratio; CI: confidence interval.

### U-Shaped FFA-MACCE risk observed only in diabetic patients

**Figure 3:**
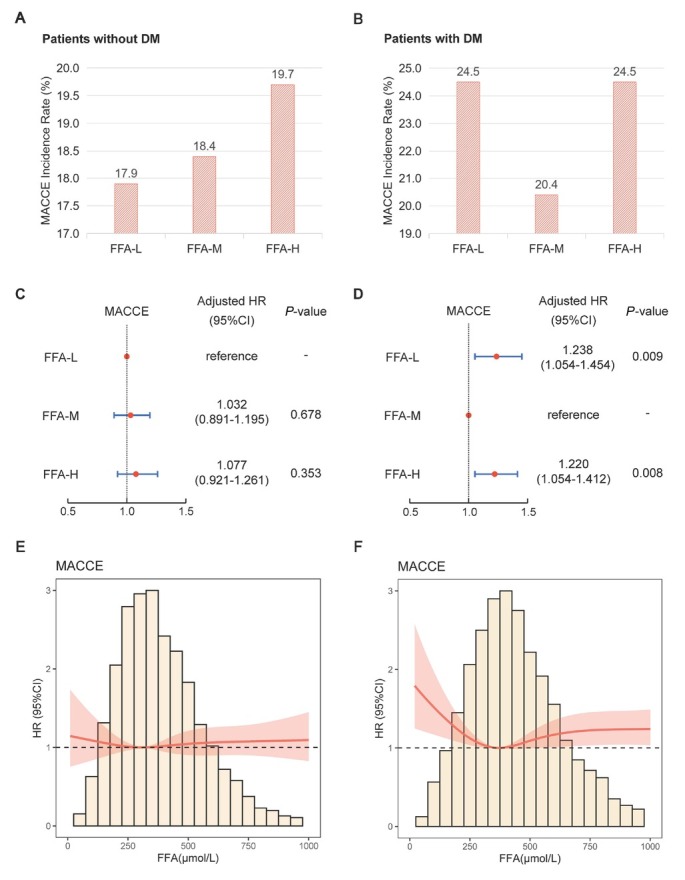
(A, B) Histograms of MACCE incidence by FFA groups; (C, D) Forest plots of MACCE by FFA groups; (E, F) Restricted cubic spline curves of FFA for MACCE. (A, C, E) Patients without DM; (B, D, F) Patients with DM. In Figures 3C and 3E, the model was adjusted for gender, age, previous myocardial infarction, previous percutaneous coronary intervention, previous coronary artery bypass grafting, hypertension, hyperlipidemia, cerebrovascular disease, chronic obstructive pulmonary disease, creatinine, blood urea nitrogen, albumin, high-sensitivity C-reactive protein, left ventricular ejection fraction, SYNTAX score, triple-vessel disease, successful previous percutaneous coronary intervention, and calcium channel blocker at discharge. In Figures 3D and 3F, the model was further adjusted to incorporate additional diabetes-related covariates (duration, therapy). FFA: free fatty acid; DM: diabetes mellitus; MACCE: major adverse cardiovascular and cerebrovascular events; HR: hazard ratio; CI, confidence interval.

Among patients without diabetes, no significant difference was observed for the risk of MACCE across the different FFA groups (Figures 3A and 3C). Among patients with diabetes, both the FFA-L and FFA-H groups had a significantly higher risk of MACCE than the FFA-M group (adjusted HR, 1.238, 95% CI 1.054–1.454, *P* = 0.009; and adjusted HR, 1.220, 95% CI, 1.054–1.412, *P* = 0.008, respectively) (Figures 3B and 3D; Figure S1). A significant interaction between DM and FFA-M group (*vs*. FFA-L group) was observed (*P* = 0.019) (Supplementary Table S4). The RCS curve exhibited a nonlinear U-shaped relationship between FFA levels and the risk of MACCE in patients with diabetes, with the lowest risk at an FFA level of 372 μmol/L (Figure 3F). In the mediation analysis, FFA only mediated 2.33% in the association between DM and MACCE risk (Supplementary Table S5).

Further analysis was performed by dividing patients into six groups based on their diabetes status and FFA levels. The non-DM/FFA-L group had the lowest incidence of MACCE (Table 3). Kaplan-Meier curves revealed that the DM/FFA-L and DM/FFA-H groups had significantly higher cumulative incidences of MACCE, followed by DM/FFA-M and non-DM/FFA-H groups, while the non-DM/FFA-L and non-DM/FFA-M groups exhibit the lowest event rate (*P* < 0.001) (Figure 2C). The DM/FFA-L and DM/FFA-H groups had significantly higher risks of MACCE than the non-DM/FFA-L group (adjusted HR 1.303, 95% CI, 1.118–1.518, *P* = 0.001 and adjusted HR 1.257, 95% CI, 1.090–1.448, *P* = 0.002, respectively) (Table 3).

**Table 3:** Univariate and multivariate Cox model analysis for endpoint events as grouped by FFA and diabetes status

Variables	Event/total (%)	Univariate analysis			Multivariate analysis		
		Crude HR	95%CI	*P* value	Adjusted HR 95%CI		*P* value
MACCE							
Non-DM/FFA-L	385/2153 (17.9)	Ref	-	-	Ref	-	-
Non-DM/FFA-M	346/1885 (18.4)	1.030	0.891–1.191	0.688	1.036	0.896–1.199	0.632
Non-DM/FFA-H	314/1590 (19.7)	1.118	0.963–1.297	0.143	1.102	0.945–1.284	0.215
DM/FFA-L	294/1202 (24.5)	1.425	1.224–1.658	< 0.001^*^	1.303	1.118–1.518	0.001^*^
DM/FFA-M	317/1554 (20.4)	1.154	0.994–1.338	0.060	1.043	0.897–1.213	0.580
DM/FFA-H	452/1846 (24.5)	1.420	1.239–1.627	< 0.001^*^	1.257	1.090–1.448	0.002^*^
All-cause death							
Non-DM/FFA-L	54/2153 (2.5)	Ref	-	-	Ref	-	-
Non-DM/FFA-M	53/1885 (2.8)	1.127	0.772–1.647	0.535	1.195	0.817–1.749	0.358
Non-DM/FFA-H	59/1590 (3.7)	1.491	1.031–2.156	0.034^*^	1.576	1.080–2.301	0.018^*^
DM/FFA-L	55/1202 (4.6)	1.850	1.271–2.693	0.001^*^	1.545	1.058–2.257	0.024^*^
DM/FFA-M	51/1554 (3.3)	1.314	0.896–1.927	0.161	1.149	0.780–1.692	0.482
DM/FFA-H	98/1846 (5.3)	2.156	1.547–3.006	< 0.001^*^	1.850	1.307–2.617	0.001^*^
Myocardial infarction							
Non-DM/FFA-L	111/2153 (5.2)	Ref	-	-	Ref	-	-
Non-DM/FFA-M	84/1885 (4.5)	0.866	0.652–1.149	0.318	0.854	0.642–1.136	0.278
Non-DM/FFA-H	87/1590 (5.5)	1.073	0.810–1.420	0.624	1.054	0.790–1.407	0.719
DM/FFA-L	69/1202 (5.7)	1.132	0.838–1.529	0.418	1.058	0.781–1.431	0.717
DM/FFA-M	94/1554 (6.0)	1.182	0.898–1.555	0.234	1.066	0.806–1.409	0.654
DM/FFA-H	104/1846 (5.6)	1.115	0.853–1.457	0.425	0.994	0.752–1.313	0.964
Unplanned revascularization							
Non-DM/FFA-L	259/2153 (12.0)	Ref	-	-	Ref	-	-
Non-DM/FFA-M	240/1885 (12.7)	1.068	0.896–1.273	0.465	1.051	0.881–1.254	0.579
Non-DM/FFA-H	197/1590 (12.4)	1.039	0.864–1.251	0.683	1.007	0.832–1.217	0.947
DM/FFA-L	184/1202 (15.3)	1.306	1.081–1.578	0.006^*^	1.237	1.023–1.497	0.028^*^
DM/FFA-M	212/1554 (13.6)	1.143	0.953–1.370	0.150	1.049	0.873–1.261	0.609
DM/FFA-H	277/1846 (15.0)	1.287	1.086–1.524	0.004^*^	1.169	0.980–1.395	0.083
Ischemic stroke							
Non-DM/FFA-L	54/2153 (2.5)	Ref	-	-	Ref	-	-
Non-DM/FFA-M	48/1885 (2.5)	1.019	0.691–1.503	0.925	1.062	0.719–1.570	0.761
Non-DM/FFA-H	41/1590 (2.6)	1.036	0.690–1.555	0.865	1.079	0.712–1.635	0.720
DM/FFA-L	52/1202 (4.3)	1.767	1.207–2.586	0.003^*^	1.570	1.070–2.306	0.021^*^
DM/FFA-M	39/1554 (2.5)	1.006	0.666–1.518	0.979	0.926	0.610–1.404	0.717
DM/FFA-H	72/1846 (3.9)	1.593	1.119–2.266	0.010^*^	1.428	0.987–2.067	0.059

* *P* value < 0.05. The multivariate Cox model was adjusted for gender, age, previous myocardial infarction, previous percutaneous coronary intervention, previous coronary artery bypass grafting, hypertension, hyperlipidemia, cerebrovascular disease, chronic obstructive pulmonary disease, creatinine, blood urea nitrogen, albumin, high-sensitivity C-reactive protein, left ventricular ejection fraction, SYNTAX score, triple-vessel disease, successful previous percutaneous coronary intervention, and calcium channel blocker at discharge. FFA: free fatty acid; MACCE: major adverse cardiac and cerebrovascular events; HR:, hazard ratio; CI: confidence interval.

In terms of secondary endpoints, compared with the non-DM/FFA-L group, the DM/FFA-L group had a significantly higher risk of all-cause death (adjusted HR 1.545, 95% CI 1.058–2.257, *P* = 0.024), unplanned revascularization (adjusted HR 1.237, 95% CI, 1.023–1.497, *P* = 0.028), and ischemic stroke (adjusted HR 1.570, 95% CI, 1.070–2.306, *P* = 0.021). In contrast, no increased risk was observed in the DM/FFA-M group across any of the endpoints after multivariable adjustment. The non-DM/FFA-H group showed a significantly higher risk of all-cause death (adjusted HR 1.576, 95% CI 1.080–2.301, *P* = 0.018), but no excess risk for other endpoints. Notably, the DM/FFA-H group exhibited the greatest risk, with all-cause death significantly higher than the non-DM/FFA-L group (adjusted HR 1.850, 95% CI 1.307–2.617, *P* = 0.001), alongside a consistent trend toward increased unplanned revascularization and ischemic stroke. (Table 3).

### Consistency of findings across clinical subgroups and sensitivity analysis

**Figure 4:**
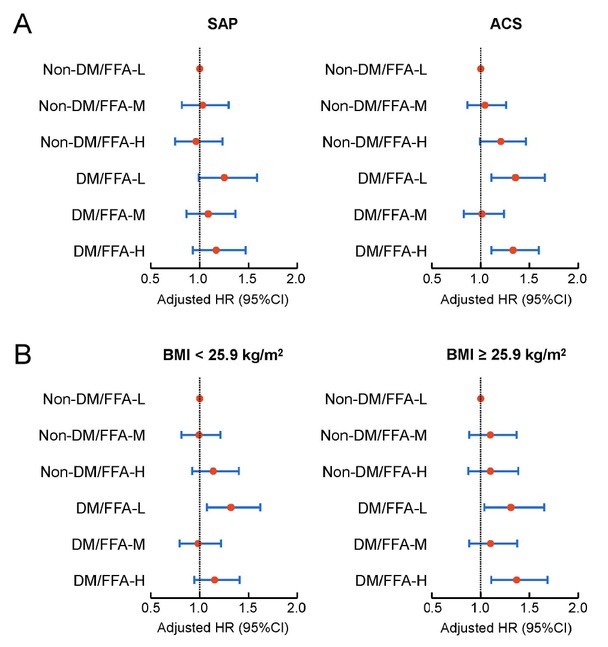
Subgroup analyses of different clinical presentations and BMI levels. Forest plots of MACCE by FFA groups in the SAP and ACS subgroups (A), and in the BMI < 25.9 kg/m^2^ and BMI ≥ 25.9 kg/m^2^ subgroups (B). In the subgroup analysis, the model was adjusted for gender, age, previous myocardial infarction, previous percutaneous coronary intervention, previous coronary artery bypass grafting, hypertension, hyperlipidemia, cerebrovascular disease, chronic obstructive pulmonary disease, creatinine, blood urea nitrogen, albumin, high-sensitivity C-reactive protein, left ventricular ejection fraction, SYNTAX score, triple-vessel disease, successful previous percutaneous coronary intervention, and calcium channel blocker at discharge. SAP: stable angina pectoris; ACS: acute coronary syndrome; BMI: body mass index; FFA: free fatty acid; DM: diabetes mellitus; MACCE: major adverse cardiovascular and cerebrovascular events; HR: hazard ratio; CI: confidence interval.

Subgroup analyses were conducted based on different clinical presentations and BMI. Patients were divided into stable angina pectoris (SAP) and ACS subgroups based on their clinical presentation. The analysis revealed no significant interaction between clinical presentation and FFA levels (*P* = 0.473). Patients were also divided into subgroups based on the median BMI (BMI < 25.9 kg/m^2^ and BMI ≥ 25.9 kg/m^2^). The results revealed no significant interaction between BMI and FFA levels (*P* = 0.791). Cox regression analysis of each subgroup yielded results consistent with the primary findings (Figure 4). Furthermore, within the patients with diabetes, we performed a stratified analysis based on insulin use. The multivariate Cox regression results, shown in Supplementary Figure S2, also indicated no significant interaction between insulin use and FFA levels (*P* = 0.221). Finally, to address the potential impact of missing data and bolster the robustness of our conclusions, we conducted a sensitivity analysis. This analysis involved performing multivariate Cox regression after using median imputation for missing FFA, HbA1c, and FBG values. The results, presented in Supplementary Table S6, were consistent with the primary findings of our study.

## Discussion

In this study, we investigated the relationship between FFA levels and long-term adverse outcomes in patients who underwent PCI. The FFA levels exhibited a nonlinear U-shaped relationship with the risk of MACCE in patients with diabetes. However, in patients without diabetes, there was no significant correlation between FFA levels and MACCE risk. The lower risk observed in diabetic patients with moderate FFA levels appears to attenuate, but not fully neutralize, and the excess MACCE risk was associated with diabetes. Our study suggested that in CAD patients with diabetes undergoing PCI, both low and high FFA levels are associated with poor prognosis. Therefore, assessing FFA levels in patients undergoing PCI with diabetes could help identify high-risk patients.

Diabetes and CAD are major health threats worldwide. Patients with CAD and comorbid diabetes have significantly worse prognoses than those without diabetes.^[20,21]^ A hallmark of this population is alterations in glucose and lipid metabolism.^[22]^ As the primary energy substrate for myocardial cells, FFAs are therefore important metabolic biomarkers.^[2]^ Several studies have demonstrated that elevated FFA levels are associated with adverse outcomes. For example, Pilz *et al*. followed 3,315 patients with CAD for 5.38 years and found that elevated FFA levels were associated with significantly higher all-cause and cardiovascular mortality.^[7]^ Similarly, in our previous study involving 15,849 patients with CAD and obtained similar results, we observed that elevated FFA levels were correlated with all-cause mortality, cardiac mortality, and major adverse cardiovascular events.^[9]^ Incorporating FFA levels into traditional risk factors has been shown to improve the accuracy of risk stratification.

However, the current evidence on the relationship between FFA levels and adverse outcomes in patients with CAD and diabetes remained limited and inconsistent. Jin *et al*. conducted a study of 5,443 patients with stable CAD and demonstrated that baseline FFA levels were associated with progressively poor prognosis, while the lowest risk was seen in patients with the lowest FFA levels.^[17]^ Given that their study population only included patients with stable CAD, we performed a subgroup analysis stratified by different clinical presentations (SAP *vs*. ACS) in our study. We found that, although the results in the SAP subgroup were not as strong as those in the ACS subgroup, the DM/FFA-L group in the SAP subgroup had a higher risk of MACCE than the DM/FFA-M group. Our findings are consistent with the work of Pan *et al*., who included 10,395 patients with CAD with a median follow-up of 24 months.^[18]^ They reported a U-shaped association between FFA levels and risks of mortality and MACCE among patients with diabetes, but not among those without diabetes. Among patients without diabetes, those with the lowest FFA levels had the lowest mortality risk. For MACCE risk, although the group with the lowest FFA levels among the patients without diabetes did not have the lowest risk of MACCE, and the differences among groups were not significant. Our study, with a longer median follow-up of 5 years, similarly supports a nonlinear U-shaped relationship between FFA levels and long-term adverse outcomes in patients with CAD and diabetes. These results suggest that assessing FFA levels in patients with CAD and diabetes can help clinicians identify high-risk groups.

Mechanistically, insulin resistance in diabetes impairs glycogen synthesis and protein metabolism in skeletal muscles, and inhibits lipoprotein lipase activity in adipocytes, leading to an increased release of FFAs.^[23]^ During acute myocardial ischemia, patients experience a stress response characterized by increased catecholamine activity, which stimulates lipolysis in the adipose tissue and elevates circulating FFA levels.^[24]^ Excess FFA levels can increase oxidative stress, promote inflammation, activate the renin-angiotensin system, disrupt nitric oxide production and release mediated by calcium signaling, ultimately leading to endothelial dysfunction.^[3,25,26,27,28]^ When myocardial uptake of fatty acids exceeds mitochondrial utilization capacity, lipid and toxic metabolite accumulation ensues, resulting in mitochondrial dysfunction and cardiac lipotoxicity.^[29,30]^

However, the mechanisms underlying the association between low FFA levels and poor prognosis in patients with diabetes and CAD remain unclear, with limited research on the subject. Under physiological conditions, myocardial cells can switch between fatty acids and glucose depending on energy demands, which is known as “metabolic flexibility”.^[31,32]^ In the presence of insulin resistance, metabolic flexibility is impaired, and myocardial cells rely predominantly on fatty acids as their energy source.^[22,23]^ Consequently, excessively low FFA levels may cause myocardial energy deficits in myocardial cells, predisposing patients to adverse cardiovascular events.^[33]^ The clinical relevance of this energy deficit is clearly demonstrated during hypoglycemia, a common complication of glucose-lowering treatments. A normal counter-regulatory response to hypoglycemia involves a rapid increase in catecholamines that stimulates lipolysis, increasing FFA availability to fuel the heart.^[34]^ Winhofer *et al*. demonstrated that during hypoglycemia, pharmacologic suppression of lipolysis with acipimox acutely reduced plasma FFA levels, accompanied by a decrease in the cardiac ejection fraction and myocardial lipid content.^[35]^ This indicates that an adequate supply of FFAs is vital for maintaining cardiac function during hypoglycemia. Therefore, low baseline FFA levels could signify an impaired ability to cardiac contractile response, leaving the heart vulnerable, and further resulting in the increased cardiovascular events. Finally, it is also plausible that extremely low FFA levels serve as a biomarker for a broader pathological state of malnutrition and frailty, which is the more direct cause of the increased cardiovascular risk.^[36]^ Further research is needed to fully elucidate the mechanism and suggest the future therapeutic strategies aiming at the normalization of FFA metabolism.

Our study supports FFA as a promising biomarker for risk stratification in CAD patients with DM and highlights the importance of maintaining FFA levels within a healthy range to balance their dual roles as energy substrates and signaling molecules. While novel pharmacotherapies targeting FFA receptors represent an important future direction, dietary modification remains the most immediate and effective intervention.^[37,38]^ Specifically, replacing harmful dietary fats with cardioprotective ones should be regarded not merely as adjunctive lifestyle advice, but as a primary, evidence-based therapeutic strategy, particularly in patients with DM.^[37,39]^ Further prospective studies and clinical trials are warranted to determine whether interventions aimed at modulating FFA levels can translate into improved long-term cardiovascular outcomes.

This study had several limitations. First, this was a single-center observational study conducted in China, and the generalizability of our findings should be validated in other racial populations. Although most confounding factors were adjusted for in the multivariate regression, residual confounding variables that were not accounted for may still exist, potentially not fully eliminating the confounding effects. Second, FFA was measured only once during hospitalization in our study. Dynamic changes in FFA levels before and after PCI were not captured, which may better reflect the correlation between FFA levels and prognosis. Third, the FFA subtypes of FFAs were not assessed in our study, preventing a precise evaluation of the association between specific FFA types and adverse outcomes. Finally, further large-scale, prospective, multicenter, and multiethnic studies are required to clarify whether maintaining FFAs within an optimal range improves prognosis and to elucidate the mechanisms underlying the association between low FFAs and adverse outcomes in patients with CAD and diabetes.

In patients with diabetes undergoing PCI, both elevated and decreased FFA levels were significantly associated with an increased risk of MACCE. However, this relationship was not observed in patients without diabetes. Our study highlights the importance of monitoring the FFA levels in patients with diabetes undergoing PCI.

## Supplementary Material

Supplementary Material Details
