# Supplementary material for: Association between free fatty acids and adverse outcomes in patients with and without diabetes undergoing percutaneous coronary intervention
Source: J Transl Int Med. 2026 Feb 13;14(1):53–63. doi: 10.1515/jtim-2026-0016 (PMC12916277; doi:10.1515/jtim-2026-0016)
Supplement: Supplementary file 1 — Supplementary Material Details [file jtim-2026-0016_sm.pdf]

Supplementary materials

Association between free fatty acids and adverse outcomes in patients with and without diabetes undergoing percutaneous coronary intervention

Qinxue Li[1#], Guyu Zeng[2#], Deshan Yuan[2], Tianyu Li[2], Peizhi Wang[2], Ce Zhang[2], Sida Jia[2], Pei Zhu[2], Ying Song[2], Xiaofang Tang[2], Ping Liu[2], Yuejin Yang[2], Runlin Gao[2], Jingjing Xu[2], Xueyan Zhao[2], Jinqing Yuan[2]

[1]Department of Cardiology, Xuanwu Hospital, Capital Medical University, National Clinical Research Centre for Geriatric Diseases, Beijing, China;

[2]Department of Cardiology, National Clinical Research Center for Cardiovascular Diseases, State Key Laboratory of Cardiovascular Disease, Fuwai Hospital, National Center for Cardiovascular Diseases, Chinese Academy of Medical Sciences and Peking Union Medical College, Beijing, China

[#]These authors contributed equally as co-first authors.

Address for Correspondence: Jinqing Yuan, Department of Cardiology, National Clinical Research Center for Cardiovascular Diseases, State Key Laboratory of Cardiovascular Disease, Fuwai Hospital, National Center for Cardiovascular Diseases, Chinese Academy of Medical Sciences and Peking Union Medical College, No 167, Beilishi Road, Xicheng District, Beijing 100037, China. Email: dr_jinqingyuan@sina.com. https://orcid.org/0000-0002-3740-5103

Supplementary information

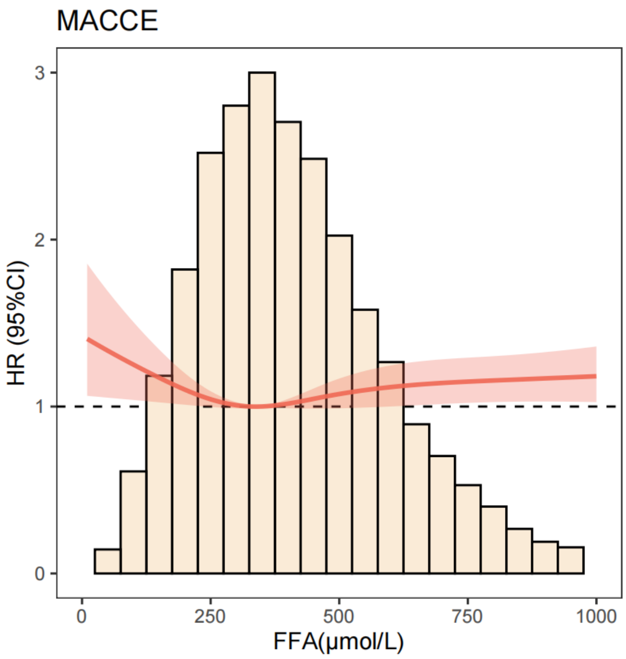

Supplementary Figure S1: Restricted cubic spline curves of FFA for MACCE in the total population. FFA, free fatty acid; MACCE, major adverse cardiovascular and cerebrovascular events. The multivariate cox model was adjusted for sex, age, previous myocardial infarction, previous percutaneous coronary intervention, previous coronary artery bypass grafting, hypertension, hyperlipidemia, cerebrovascular disease, chronic obstructive pulmonary disease, creatinine, blood urea nitrogen, albumin, high-sensitivity C-reactive protein, left ventricular ejection fraction, SYNTAX score, triple-vessel disease, successful previous percutaneous coronary intervention, and calcium channel blocker at discharge.

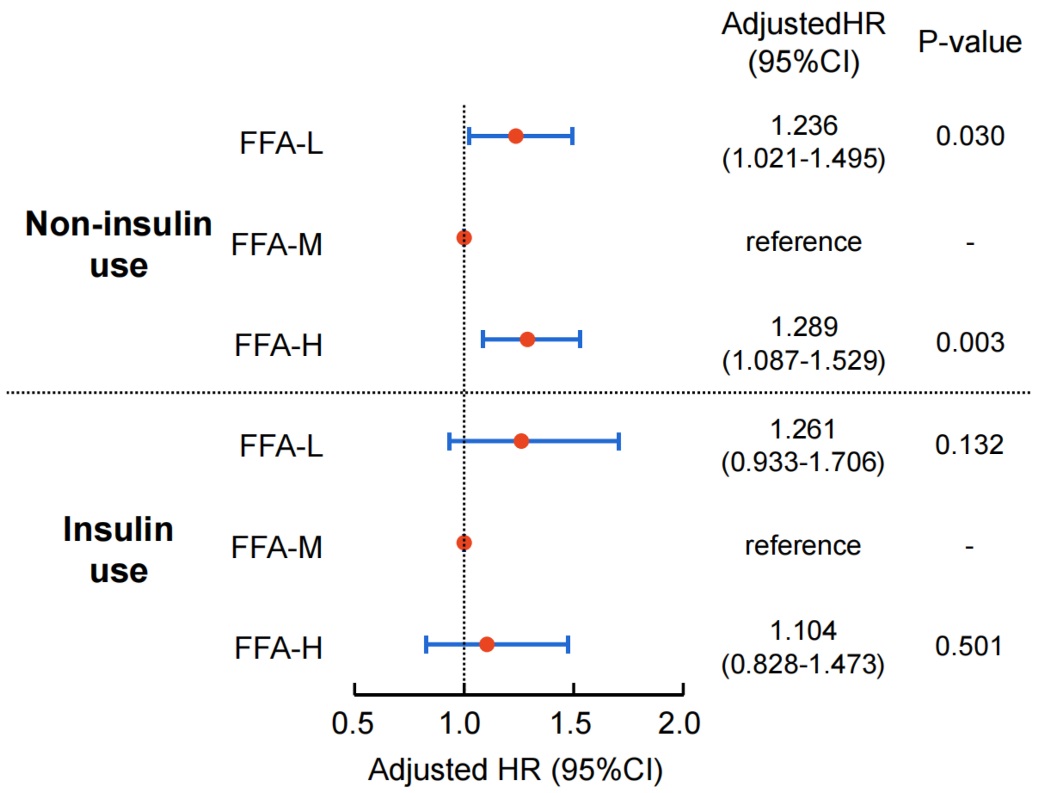

Supplementary Figure S2: The association between FFA and MACCE, stratified by insulin use in patients with diabetes undergoing PCI. FFA, free fatty acid; MACCE, major adverse cardiovascular and cerebrovascular events; HR, hazard ratio; CI, confidence interval. The multivariate cox model was adjusted for sex, age, previous myocardial infarction, previous percutaneous coronary intervention, previous coronary artery bypass grafting, hypertension, hyperlipidemia, cerebrovascular disease, chronic obstructive pulmonary disease, creatinine, blood urea nitrogen, albumin, high-sensitivity C-reactive protein, left ventricular ejection fraction, SYNTAX score, triple-vessel disease, successful previous percutaneous coronary intervention, and calcium channel blocker at discharge.

Supplementary Table S1: Complete results of univariate and multivariate cox model analysis of MACCE for all participants.

	Univariate model			Multivariate model		
	**Crude HR**	**95%CI**	***P* value**	**Adjusted HR**	**95%CI**	***P* value**
FFA						
FFA-L	1.058	0.950-1.177	0.305	1.085	0.973-1.209	0.141
FFA-M	Ref	-	-	Ref	-	-
FFA-H	1.177	1.061-1.306	0.002*	1.131	1.017-1.257	0.023*
Diabetes	1.275	1.170-1.388	<0.001*	1.144	1.047-1.250	0.003*
Male	1.060	0.956-1.175	0.270	1.099	0.978-1.236	0.114
Age	1.012	1.008-1.016	<0.001*	1.007	1.002-1.012	0.003*
Previous MI	1.210	1.092-1.341	<0.001*	1.049	0.938-1.172	0.404
Previous PCI	1.353	1.233-1.485	<0.001*	1.247	1.128-1.378	<0.001*
Hypertension	1.210	1.104-1.326	<0.001*	1.102	1.001-1.214	0.048*
Hyperlipidaemia	1.093	0.997-1.199	0.058	1.026	0.934-1.128	0.592
Previous CABG	1.380	1.141-1.669	0.001*	1.084	0.888-1.323	0.427
CVD	1.289	1.135-1.463	<0.001*	1.140	1.001-1.298	0.049*
COPD	1.359	1.060-1.743	0.016*	1.285	0.999-1.652	0.051
Creatine	1.006	1.003-1.009	<0.001*	1.002	0.999-1.005	0.256
BUN	1.052	1.026-1.077	<0.001*	1.013	0.985-1.041	0.364
Albumin	0.990	0.979-1.000	0.055	0.999	0.987-1.011	0.851
hsCRP	1.020	1.009-1.031	<0.001*	1.013	1.002-1.025	0.023*
LVEF<50%	1.201	1.033-1.396	0.017*	1.094	0.937-1.278	0.254
SYNTAX score	1.016	1.011-1.021	<0.001*	1.006	1.001-1.012	0.021*

	**Univariate model**			**Multivariate model**		
	**Crude HR**	**95%CI**	***P* value**	**Adjusted HR**	**95%CI**	***P* value**
TVD	1.588	1.458-1.730	<0.001*	1.405	1.281-1.542	<0.001*
Successful PCI	0.571	0.479-0.682	<0.001*	0.648	0.541-0.776	<0.001*
CCB at discharge	1.077	0.989-1.173	0.089	1.060	0.970-1.157	0.198
ACS	1.029	0.943-1.123	0.526			
BMI	0.999	0.986-1.013	0.932			
Family history of CAD	1.015	0.920-1.121	0.765			
PAD	1.139	0.884-1.466	0.314			
Current smoker	1.066	0.977-1.162	0.151			
HDL-C	0.884	0.757-1.033	0.122			
LDL-C	1.029	0.982-1.078	0.227			
TG	1.015	0.976-1.055	0.463			
Left main involved	1.098	0.928-1.298	0.276			
Number of stents	1.001	0.963-1.041	0.951			
Aspirin at discharge	0.819	0.575-1.168	0.271			
Clopidogrel at discharge	1.060	0.735-1.530	0.755			
β-blocker at discharge	1.030	0.891-1.191	0.689			
Statin at discharge	0.947	0.766-1.171	0.616			
Nitrate at discharge	1.191	0.864-1.642	0.285			

* means *P* value < 0.05

FFA, free fatty acid; MI, myocardial infarction; PCI, percutaneous coronary intervention; CABG, coronary artery bypass grafting; CVD, cerebrovascular disease; COPD, chronic obstructive pulmonary disease; BUN, blood urea nitrogen; hsCRP,

high-sensitivity C-reactive protein; LVEF, left ventricular ejection fraction; SYNTAX, synergy between PCI with taxus and cardiac surgery; TVD, triple-vessel disease; CCB, calcium channel blocker; ACS, acute coronary syndrome; BMI, body mass index; CAD, coronary artery disease; PAD, peripheral vascular disease; HDL-C, high-density lipoprotein cholesterol; LDL-C, low-density lipoprotein cholesterol; TG, triglyceride. The multivariate Cox model was adjusted for sex, age, previous myocardial infarction, previous percutaneous coronary intervention, previous coronary artery bypass grafting, hypertension, hyperlipidemia, cerebrovascular disease, chronic obstructive pulmonary disease, creatinine, blood urea nitrogen, albumin, high-sensitivity C-reactive protein, left ventricular ejection fraction, SYNTAX score, triple-vessel disease, successful previous percutaneous coronary intervention, and calcium channel blocker at discharge.

Supplementary Table S2: Baseline characteristics of different FFA groups of patients without diabetes.

**Variables**	**Non-DM/FFA-L (*n* = 2153)**	**Non-DM/FFA-M (*n* = 1885)**	**Non-DM/FFA-H (*n* = 1590)**	***P* value**
Demographic characteristics				
Age, years	57.62 ± 10.27	56.88 ± 10.57	57.80 ± 10.57	0.020*
Male, %	1761 (81.8)	1524 (80.8)	1188 (74.7)	<0.001*
BMI, kg/m^2	25.04 ± 3.01	25.97 ± 3.08	26.08 ± 3.42	<0.001*
Clinical presentation				
SAP, %	855 (39.7)	729 (38.7)	608 (38.2)	0.630
ACS, %	1298 (60.3)	1156 (61.3)	982 (61.8)	
Coexisting conditions				
Previous MI, %	402 (18.7)	354 (18.8)	258 (16.2)	0.090

Variables	Non-DM/FFA-L (*n* = 2153)	Non-DM/FFA-M (*n* = 1885)	Non-DM/FFA-H (*n* = 1590)	*P* value
Previous PCI, %	465 (21.6)	413 (21.9)	318 (20.0)	0.345
Previous CABG, %	63 (2.9)	66 (3.5)	62 (3.9)	0.254
Hypertension, %	1205 (56.0)	1179 (62.5)	1016 (63.9)	<0.001 *
Hyperlipidaemia, %	1338 (62.1)	1240 (65.8)	992 (62.4)	0.034*
Family history of CAD, %	486 (22.6)	467 (24.8)	442 (27.8)	0.001*
CVD, %	216 (10.0)	173 (9.2)	123 (7.7)	0.054
PAD, %	41 (1.9)	33 (1.8)	35 (2.2)	0.094
COPD, %	51 (2.4)	33 (1.8)	40 (2.5)	0.248
Current smoker, %	1314 (61.0)	1112 (59.0)	859 (54.0)	<0.001 *
Laboratory measurements				
FFA, µmol/L	222.80 ± 64.36	389.28 ± 45.72	639.20 ± 176.28	<0.001 *
HDL-C, mmol/L	1.03 ± 0.26	1.02 ± 0.28	1.10 ± 0.31	<0.001 *
LDL-C, mmol/L	2.42 ± 0.88	2.51 ± 0.90	2.63 ± 0.94	<0.001 *
TG, mmol/L	1.56 ± 0.77	1.76 ± 0.95	1.81 ± 1.11	<0.001 *

Variables	Non-DM/FFA-L (*n* = 2153)	Non-DM/FFA-M (*n* = 1885)	Non-DM/FFA-H (*n* = 1590)	*P* value
FBG, mmol/L	5.04 ± 0.53	5.11 ± 0.54	5.29 ± 0.62	<0.001*
HbA1c, %	5.88 ± 0.34	5.88 ± 0.33	5.88 ± 0.34	0.932
Creatine, umol/l	75.05 ± 14.35	75.33 ± 14.22	75.71 ± 15.42	0.393
BUN, mmol/L	5.81 ± 1.55	5.71 ± 1.51	5.52 ± 1.56	<0.001*
Albumin, g/L	41.83 ± 3.79	42.90 ± 3.96	44.37 ± 4.00	<0.001*
hsCRP, mg/L	2.62 ± 3.36	3.02 ± 3.66	3.32 ± 3.89	<0.001*
Cardiac function				
LVEF<50%, %	138 (6.4)	141 (7.5)	131 (8.2)	0.096
Angiographic and procedural details				
Left main involved, %	131 (6.1)	105 (5.6)	89 (5.6)	0.735
TVD, %	796 (37.0)	693 (36.8)	630 (39.6)	0.158
SYNTAX score	11.26 ± 7.89	11.02 ± 7.47	11.82 ± 8.10	0.009*
Successful PCI, %	2069 (96.1)	1814 (96.2)	1522 (95.7)	0.732
Number of stents	1.76 ± 1.08	1.79 ± 1.07	1.78 ± 1.05	0.773
Medicine at discharge				

Variables	Non-DM/FFA-L (*n* = 2153)	Non-DM/FFA-M (*n* = 1885)	Non-DM/FFA-H (*n* = 1590)	*P* value
Aspirin, %	2123 (98.6)	1861 (98.7)	1575 (99.1)	0.453
Clopidogrel, %	2113 (98.1)	1853 (98.3)	1574 (99.0)	0.098
CCB, %	985 (45.8)	856 (45.4)	806 (50.7)	0.003*
β-blocker, %	1892 (87.9)	1688 (89.5)	1431 (90.0)	0.083
Statin, %	2073 (96.3)	1827 (96.9)	1536 (96.6)	0.536
Nitrate, %	2109 (98.0)	1853 (98.3)	1558 (98.0)	0.690

* means *P* value < 0.05
FFA, free fatty acid; BMI, body mass index; SAP, stable angina pectoris; ACS, acute coronary syndrome; MI, myocardial infarction; PCI, percutaneous coronary intervention; CABG, coronary artery bypass grafting; CAD, coronary artery disease; CVD, cerebrovascular disease; PAD, peripheral vascular disease; COPD, chronic obstructive pulmonary disease; DM, diabetes mellitus; HDL-C, high-density lipoprotein cholesterol; LDL-C, low-density lipoprotein cholesterol; TG, triglyceride; FBG, fasting blood glucose; HbA1c, glycated hemoglobin; BUN, blood urea nitrogen; hsCRP, high-sensitivity C-reactive protein; LVEF, left ventricular ejection fraction; TVD, triple-vessel disease; SYNTAX, synergy between PCI with taxus and cardiac surgery; CCB, calcium channel blocker.

Supplementary Table S3: Baseline characteristics of different FFA groups of patients with diabetes.

Variables	DM/FFA-L (*n* = 1202)	DM/FFA-M (*n* = 1554)	DM/FFA-H (*n* = 1846)	*P* value
Demographic characteristics				
Age, years	59.34 ± 9.61	59.01 ± 9.85	60.07 ± 10.23	0.007*
Male, %	934 (77.7)	1196 (77.0)	1291 (69.9)	<0.001*
BMI, kg/m^2	25.77 ± 2.94	26.45 ± 3.17	26.50 ± 3.23	<0.001*

Variables	DM/FFA-L (*n* = 1202)	DM/FFA-M (*n* = 1554)	DM/FFA-H (*n* = 1846)	*P* value
Clinical presentation				
SAP, %	519 (43.2)	683 (44)	688 (37.3)	<0.001*
ACS, %	683 (56.8)	871 (56.0)	1158 (62.7)	
Coexisting conditions				
Previous MI, %	271 (22.5)	333 (21.4)	345 (18.7)	0.023*
Previous PCI, %	314 (26.1)	455 (29.3)	547 (29.6)	0.085
Previous CABG, %	62 (5.2)	74 (4.8)	86 (4.7)	0.813
Hypertension, %	766 (63.7)	1083 (69.7)	1353 (73.3)	<0.001*
Hyperlipidaemia, %	861 (71.6)	1119c (72.0)	1324 (71.7)	0.973
Family history of CAD, %	291 (24.2)	374 (24.1)	472 (25.6)	0.538
CVD, %	168 (14.0)	191 (12.3)	222 (12.0)	0.253
PAD, %	54 (4.5)	47 (3.0)	61 (3.3)	0.094
COPD, %	29 (2.4)	42 (2.7)	40 (2.2)	0.598
Current smoker, %	710 (59.1)	920 (59.2)	936 (50.7)	<0.001*
Laboratory measurements				
FFA, μmol/L	222.99 ± 66.09	393.82 ± 45.85	697.65 ± 269.05	<0.001*
HDL-C, mmol/L	0.99 ± 0.25	0.98 ± 0.25	1.06 ± 0.29	<0.001*
LDL-C, mmol/L	2.38 ± 0.82	2.46 ± 0.90	2.63 ± 0.97	<0.001*
TG, mmol/L	1.63 ± 0.92	1.89 ± 1.07	2.04 ± 1.44	<0.001*

Variables	DM/FFA-L (*n* = 1202)	DM/FFA-M (*n* = 1554)	DM/FFA-H (*n* = 1846)	*P* value
FBG, mmol/L	6.98 ± 2.35	7.22 ± 2.34	7.93 ± 2.67	<0.001*
HbA1c, %	7.40 ± 1.30	7.53 ± 1.32	7.60 ± 1.38	<0.001*
Creatine, umol/l	75.97 ± 16.03	75.37 ± 16.71	75.81 ± 18.18	0.632
BUN, mmol/L	6.14 ± 1.85	6.00 ± 1.66	5.92 ± 1.82	0.004*
Albumin, g/L	41.63 ± 3.77	42.82 ± 3.96	43.78 ± 4.05	<0.001*
hsCRP, mg/L	3.13 ± 3.72	3.34 ± 3.80	3.98 ± 4.27	<0.001*
Cardiac function				
LVEF<50%, %	93 (7.7)	116 (7.5)	169 (9.2)	0.158
Angiographic and procedural details				
Left main involved, %	94 (7.8)	94 (6.0)	141 (7.6)	0.116
TVD, %	544 (45.3)	746 (48.0)	900 (48.8)	0.155
SYNTAX score	12.11 ± 8.26	11.95 ± 8.53	12.29 ± 8.37	0.507
Successful PCI, %	1151 (95.8)	1494 (96.1)	1761 (95.4)	0.564
Number of stents	1.82 ± 1.11	1.90 ± 1.19	1.80 ± 1.14	0.046*
Medicine at discharge				
Aspirin, %	1194 (99.3)	1536 (98.8)	1814 (98.3)	0.032*
Clopidogrel, %	1187 (98.8)	1532 (98.6)	1820 (98.6)	0.915
CCB, %	575 (47.8)	777 (50.0)	986 (53.4)	0.008*
β-blocker, %	1108 (92.2)	1415 (91.1)	1696 (91.9)	0.527
Statin, %	1145 (95.3)	1488 (95.8)	1749 (94.7)	0.389

Variables	DM/FFA-L (*n* = 1202)	DM/FFA-M (*n* = 1554)	DM/FFA-H (*n* = 1846)	*P* value
Nitrate, %	1178 (98.0)	1517 (97.6)	1798 (97.4)	0.563
Insulin, %	324 (27.0)	377 (24.3)	490 (26.5)	0.195

* means *P* value < 0.05

FFA, free fatty acid; BMI, body mass index; SAP, stable angina pectoris; ACS, acute coronary syndrome; MI, myocardial infarction; PCI, percutaneous coronary intervention; CABG, coronary artery bypass grafting; CAD, coronary artery disease; CVD, cerebrovascular disease; PAD, peripheral vascular disease; COPD, chronic obstructive pulmonary disease; DM, diabetes mellitus; HDL-C, high-density lipoprotein cholesterol; LDL-C, low-density lipoprotein cholesterol; TG, triglyceride; FBG, fasting blood glucose; HbA1c, glycated hemoglobin; BUN, blood urea nitrogen; hsCRP, high-sensitivity C-reactive protein; LVEF, left ventricular ejection fraction; TVD, triple-vessel disease; SYNTAX, synergy between PCI with taxus and cardiac surgery; CCB, calcium channel blocker.

Supplementary Table S4: The interaction effect between DM and FFA tertiles for MACCE risks.

Variable	HR (95% CI)	*P* value
Main effects		
DM (yes *vs*. no)	1.303 (1.118-1.284)	<0.001
FFA-M *vs*. FFA-L	1.036 (0.896-1.199)	0.632
FFA-H *vs*. FFA-L	1.102 (0.946-1.284)	0.214
Interactions		
FFA-M (*vs*. FFA-L)×DM	0.772 (0.623-0.958)	**0.019**
FFA-H (*vs*. FFA-L)×DM	0.875 (0.710-1.079)	0.213

DM, diabetes mellitus; FFA, free fatty acid; MACCE, major adverse cardiac and cerebrovascular events.

Supplementary Table S5: Mediation analysis of free fatty acid in the association between diabetes mellitus and MACCE risk.

	HR (95%CI)	*P* value
Average Indirect Effect	0.996 (0.981-1.010)	0.562
Average Direct Effect	0.848 (0.761-0.945)	0.003
Total Effect	0.845 (0.759-0.940)	0.002

Prop. Mediated (%)	2.33 (-5.76-10.43)	0.572

CI, confidence interval; HR, mazard ratio; MACCE, major adverse cardiac and cerebrovascular events.

Supplementary Table S6: Multivariate cox model analysis of MACCE event after median imputation for missing FFA, HbA1c, and FBG data, grouped by FFA and diabetes status.

	MACCE		
	**Adjusted HR**	**95%CI**	***P* value**
Non-DM/FFA-L	Ref	-	-
Non-DM/FFA-M	1.065	0.927-1.222	0.374
Non-DM/FFA-H	1.108	0.953-1.288	0.181
DM/FFA-L	1.310	1.126-1.526	<0.001*
DM/FFA-M	1.047	0.902-1.217	0.545
DM/FFA-H	1.259	1.093-1.449	0.001*

* means *P* value < 0.05

FFA, free fatty acid; MACCE, major adverse cardiac and cerebrovascular events; HR, hazard ratio; CI, confidence interval. The multivariate Cox model was adjusted for sex, age, previous myocardial infarction, previous percutaneous coronary intervention, previous coronary artery bypass grafting, hypertension, hyperlipidemia, cerebrovascular disease, chronic obstructive pulmonary disease, creatinine, blood urea nitrogen, albumin, high-sensitivity C-reactive protein, left ventricular ejection fraction, SYNTAX score, triple-vessel disease, successful previous percutaneous coronary intervention, and calcium channel blocker at discharge.
